# Prediction of Cardiovascular Risk Among People with HIV Using the PREVENT Equations Compared to the Pooled Cohort Equations

**DOI:** 10.1007/s11606-025-09642-z

**Published:** 2025-06-06

**Authors:** Matthew S. Durstenfeld, Megan M. McLaughlin, Monica Gandhi, John Kornak, Alexis L. Beatty, Priscilla Y. Hsue

**Affiliations:** 1 Department of Medicine, University of California San Francisco, San Francisco, USA; 2 Zuckerberg San Francisco General, San Francisco, USA; 3 Department of Epidemiology and Biostatistics, University of California, San Francisco, San Francisco, USA; 4 Division of Cardiology, David Geffen School of Medicine at the University of California Los Angeles, Los Angeles, USA; 5 UCSF Division of Cardiology at Zuckerberg San Francisco General Hospital, 1001 Potrero Avenue, 5G8, San Francisco, CA 94110 USA

## Abstract

**Background:**

People with HIV (PWH) are at elevated risk of atherosclerotic cardiovascular disease (ASCVD), and current risk prediction tools underestimate risk among PWH. The American Heart Association developed new risk prediction equations, Predicting Risk of cardiovascular disease EVENTs (PREVENT), which have not been studied among PWH.

**Objective:**

To compare predicted 10-year ASCVD risk using PREVENT with the pooled cohort equations (PCE) and the implications for statin recommendations among PWH.

**Design:**

A cross-sectional observational study using real-world, electronic health records

**Participants:**

All people with HIV ages 40 to 75 without cardiovascular disease at 23 primary care and HIV clinics affiliated with two health systems in San Francisco, California from 2019 to 2024

**Main Measures:**

We compared predicted 10-year ASCVD using the PREVENT equations and the PCE. Then we considered implications of PREVENT for statin therapy using current guidelines.

**Key Results:**

Among 3357 PWH (median 57 years old; 73% male,12% female, 15% transgender/nonbinary/nondisclosed; 20% Black and 25% Latino), 91% were on antiretroviral therapy and 86% had virologic suppression. Among 2853 PWH with complete data for both calculators, the median predicted risk was 7.7% (interquartile range (IQR) 3.7, 14.0) using the PCE and 3.3% (IQR 1.9, 5.4) using PREVENT. Predicted risk was lower for 97% of individuals using PREVENT. Using a 10-year ASCVD risk threshold of 5%, only 28.6% of PWH would be strongly recommended for statins with PREVENT compared to 67.3% with PCE. The difference in predicted risk between the two equations varied across sex and race/ethnicity.

**Conclusions:**

The PREVENT equations predict lower 10-year ASVCD risk for PWH compared to the PCE, which underpredicts risk for PWH. Underprediction of ASCVD risk, which —using new guidelines —would translate to 58% fewer PWH strongly recommended for statins using PREVENT compared to PCE, has the potential to increase cardiovascular disease and worsen healthcare disparities among PWH.

**Supplementary Information:**

The online version contains supplementary material available at 10.1007/s11606-025-09642-z.

## INTRODUCTION

People with HIV (PWH) have 1.5- to 2-fold higher risk of cardiovascular disease (CVD) compared to people without HIV.^1^ Based on the risk-based paradigm endorsed by the US Preventive Services Task Force (USPSTF) and cardiology societies, pharmacologic risk reduction is encouraged when absolute risk reduction exceeds potential risk of harm and societal costs.^2^ The Randomized Trial to Prevent Vascular Events in HIV (REPRIEVE) provided strong evidence to support the use of statins among people with HIV at low to intermediate cardiovascular risk.^3^ In response to REPRIEVE, the United States Department of Health and Human Services (DHHS) Guidelines Panel for the Use of Antiretroviral Therapy in Adults and Adolescents with HIV favors moderate-intensity statin initiation in those with a predicted 10-year risk <5%, strongly recommends at least a moderate-intensity statin for all PWH age 40–75 with an estimated 10-year risk of 5–20%, and strongly recommends a high-intensity statin for those with risk >20%.^4,5^ These recommendations were endorsed by the American College of Cardiology, American Heart Association (AHA), and the HIV Medicine Association.

A major challenge to implementing this paradigm for PWH is poor discrimination and calibration of risk prediction models in this population.^6–8^ The pooled cohort equations (PCE)-based risk estimation, previously endorsed by the AHA, was used in REPRIEVE and is used in clinical practice in the USA. However, using PCE underestimates risk among PWH, especially among women and those who identify as Black or African American in high-income countries.^9–11^ Unfortunately, HIV-specific calculators such as the D:A:D calculator do not perform better and have yet to be externally validated in other cohorts of PWH.^7,11,12^

The AHA recently developed a set of new risk calculators collectively called “Predicting Risk of cardiovascular disease EVENTs (PREVENT^TM^)” that incorporate cardiovascular-kidney-metabolic health.^13^ They have several potential advantages, which may be highly relevant to 10-year ASCVD risk prediction to inform statin use, including incorporation of renal function and body mass index. They also trade race-based equations for a ZIP-code based social deprivation index, which may have health equity implications. PREVENT has not yet been evaluated among PWH.

Therefore, we sought to compare the 10-year ASCVD risk predicted by the PREVENT equations with the risk predicted by the PCE using a real-world electronic health record-based cohort of diverse PWH to consider the implications for statin recommendations and examine how they compare among key sex and race/ethnicity subgroups.

## METHODS

### Study Design

This study is a retrospective cross-sectional study using an electronic health record-based cohort from the University of California San Francisco Health System (UCSF Health), an academic medical center, and the San Francisco Department of Public Health (SFDPH), the municipal health system where many patients with Medicaid receive care, from 2019 to 2024. Data were extracted on November 1, 2024, and analyzed in November 2024.

### Study Population and Setting

We included individuals aged 40 to 75 years old with at least one ambulatory clinical encounter at one of 23 primary care or HIV clinics within UCSF or SFDPH from September 2019 to October 2024 who were diagnosed with HIV. The UCSF HIV Clinic serves those with a mix of private and public insurance, and the SFDPH clinics (including the Ward 86 HIV Clinic at San Francisco General Hospital) serve those with public insurance only. As in our previous work, HIV was defined by 10 th revision of the International Statistical Classification of Diseases (ICD-10) code and at least one recorded viral load to increase specificity.^14,15^ We excluded those with an ICD code for a prior cardiovascular event, including myocardial infarction, ischemic stroke, prior coronary revascularization, and heart failure, groups for which the risk calculators were not intended to be used.

### Data

Most of the variables needed to calculate predicted risk were extracted directly from the electronic health record data warehouses with two exceptions. Body mass index was calculated from height and weight. Estimated glomerular filtration rate was calculated from the serum creatinine using the CKD EPI equations via the “transplantr” R package. We used ZIP codes to calculate the social deprivation index exactly as done in the PREVENT equations without rescaling to the distribution in the San Francisco Bay Area. We used the most recent available values from the electronic health record starting in 2019. For the primary analyses, we excluded those with extreme values outside of the range of the calculators (systolic blood pressure <90 mm Hg or >200 mm Hg, diastolic blood pressure <40 mm Hg or >120 mm Hg, total cholesterol >320 mg/dL or <130 mg/dL, body mass index <18.5 kg/m^2^ or >39.9 kg/m^2^) in accordance with the intended use of the equations. In our imputed data (see missing data below), extreme values were Winsorized to the minimum or maximum allowed for the calculators (<1% of missing data).

### Outcomes

The primary outcomes were the predicted 10-year ASCVD risk using the pooled cohort equations (PCE)^16^ and the PREVENT_full_ equations.^17^ We chose to use the PCE as these are specifically recommended in guidelines, were used in the REPRIEVE study, and have been previously used in multiple studies, including among PWH. The PREVENT equations have several versions: the base model, a model which adds urine albumin creatinine ratio, a model which adds hemoglobin A1c, a model which adds social deprivation index, and a “full” model which adds all of those to the base model. We chose to use the PREVENT_full_ equations rather than the base equations as our primary outcome as recommended by the developers when those variables are available. Secondary outcomes included predicted risk using the PREVENT_base_ equations^17^ and the revised pooled cohort equations (rPCE).^18^ We used the “PooledCohort” R package to calculate risk using the pooled cohort equations (including both the original Goff 2013 version and the revised Yadlowsky 2018 version) and the “AHAprevent” R package to calculate risk using the PREVENT equations.

### Missing Data

Our primary analysis was complete case analysis using only those individuals with all the data necessary to calculate predicted risk using both the PCE and PREVENT, as this reflects the clinical use case. We conducted a sensitivity analysis including all individuals using multiple imputation for missing data (predominantly missing cholesterol and estimated glomerular filtration rate) using the “MICE” package with 10 imputations.

### Analysis

Data cleaning, analysis, and visualization were conducted using RStudio version 2024.09.0 Build 375 (“Cranberry Hibiscus”) and R version 4.4.1. We plotted the predicted risk by the two equations and compared the predicted risk using the Wilcoxon signed rank test given the non-normal distribution of differences in probabilities. To compare these by subgroups, we used the Wilcoxon rank sum test when there were two groups (sex) and the Kruskal-Wallis test when there were more than two groups (race/ethnicity). We also determined for each individual whether their predicted risk exceeded the 5% risk threshold for a strong statin recommendation and compared this between the two equations using the *χ*^2^ test. In a sensitivity analysis, we used multivariate imputation (“MICE” package) using the other covariates in the risk prediction equations as predictors.

### Human Ethics and Consent to Participate Declarations

This study received ethics approval from the UCSF Institutional Review Board (#22-37224). A waiver of consent was granted to use electronic health records of routine clinical data; no patient identifiers are ever presented in the output of our analyses.

## RESULTS

The overall population who met our inclusion criteria included 3725 individuals with HIV, of whom 2853 had complete data to calculate risk using both PCE and PREVENT. The median age of those with complete data was 57 years; 84% were male by natal sex and 74.5% were cis-gender male by preferred gender identity (Table 1). In terms of race and ethnicity, 19% with complete data identified as Black and 24% as Hispanic. Nearly all were prescribed antiretroviral therapy (89%), most achieved viral suppression on their most recent viral load measurement (84%), and the median CD4 count was 564 (IQR 382, 791). There were differences between the population with complete data and those with missing data in terms of several measured variables, including insurance status, viral load, and cardiovascular risk factors (Table 1).

**Table 1:** Participant Characteristics by Complete Data Status

	Complete data (*N*=2853)	Missing data (*N*=872)	Overall (*N*=3725)
Age, median [q1, q3], years	57 [50, 63]	55 [46, 62]	57.0 [49, 63]
Natal sex			
Male	2395 (83.9%)	730 (83.7%)	3125 (83.9%)
Female	458 (16.1%)	132 (15.1%)	590 (15.8%)
Missing	0 (0%)	10 (1.1%)	10 (0.3%)
Gender identity			
Female	379 (13.3%)	95 (10.9%)	474 (12.7%)
Male	2125 (74.5%)	522 (59.9%)	2647 (71.1%)
Transgender female	82 (2.9%)	33 (3.8%)	115 (3.1%)
Transgender male	5 (0.2%)	2 (0.2%)	7 (0.2%)
Non-binary/gender queer/other	20 (0.7%)	12 (1.4%)	32 (0.9%)
Choose not to disclose	27 (0.9%)	17 (1.9%)	44 (1.2%)
Missing	215 (7.5%)	191 (21.9%)	406 (10.9%)
Race/ethnicity			
Asian	275 (9.6%)	61 (7.0%)	336 (9.0%)
Black	537 (18.8%)	191 (21.9%)	728 (19.5%)
Hispanic/Latino	689 (24.2%)	219 (25.1%)	908 (24.4%)
Native American	87 (3.0%)	21 (2.4%)	108 (2.9%)
Pacific Islander/Hawaiian	29 (1.0%)	7 (0.8%)	36 (1.0%)
White	1466 (51.4%)	436 (50.0%)	1902 (51.1%)
Other	640 (22.4%)	209 (24.0%)	849 (22.8%)
Race missing	33 (1.2%)	10 (1.1%)	43 (1.2%)
Insurance			
Private	664 (23.3%)	154 (17.7%)	818 (22.0%)
Medicaid	1205 (42.2%)	436 (50.0%)	1641 (44.1%)
Medicare	922 (32.3%)	250 (28.7%)	1172 (31.5%)
Uninsured	58 (2.0%)	28 (3.2%)	86 (2.3%)
Veterans Affairs	2 (0.1%)	1 (0.1%)	3 (0.1%)
Missing	2 (0.1%)	3 (0.3%)	5 (0.1%)
Social Deprivation Index, median [Q1, Q3]	8 (7, 10)	8 (7, 10)	8 (7, 10)
1	8 (0.3%)	4 (0.5%)	12 (0.3%)
2	34 (1.2%)	8 (0.9%)	42 (1.1%)
3	30 (1.1%)	9 (1.0%)	39 (1.0%)
4	300 (10.5%)	86 (9.9%)	386 (10.4%)
5	104 (3.6%)	24 (2.8%)	128 (3.4%)
6	152 (5.3%)	37 (4.2%)	189 (5.1%)
7	282 (9.9%)	93 (10.7%)	375 (10.1%)
8	712 (25.0%)	247 (28.3%)	959 (25.7%)
9	158 (5.5%)	36 (4.1%)	194 (5.2%)
10	1047 (36.7%)	315 (36.1%)	1362 (36.6%)
Missing	26 (0.9%)	13 (1.5%)	39 (1.0%)
Antiretroviral therapy	2552 (89.4%)	679 (77.9%)	3231 (86.7%)
Undetectable viral load	2418 (84.8%)	588 (67.4%)	3006 (80.7%)
Viral load (among those detectable), median [Q1, Q3], Copies/mL	175 [48.0, 6700]	2430 [154, 67800]	514 [57.5, 21900]
Missing	115 (4.0%)	126 (14.4%)	241 (6.5%)
CD4, Median [Q1, Q3], Cells/mL	564 [382, 791]	473 [276, 701]	545 [358, 772]
Missing	222 (7.8%)	201 (23.1%)	423 (11.4%)
Hypertension	1462 (51.2%)	316 (36.2%)	1778 (47.7%)
Systolic blood pressure, median [Q1, Q3], mm Hg	126 [116, 136]	125 [114, 136]	126 [115, 136]
Missing	0 (0%)	17 (1.9%)	17 (0.5%)
Hyperlipidemia	1701 (59.6%)	241 (27.6%)	1942 (52.1%)
Total cholesterol, median [Q1, Q3], mg/dL^a^	169 [145, 196]	179 [148, 205]	170 [146, 198]
Missing	0 (0%)	420 (48.2%)	420 (11.3%)
NonHDL cholesterol, median [Q1, Q3], mg/dL^a^	119 [97.0, 144]	122 [99.0, 149]	119 [97.0, 145]
Missing	0 (0%)	420 (48.2%)	420 (11.3%)
LDL cholesterol, median [Q1, Q3], mg/dL^a^	92.0 [71.0, 114]	97.0 [76.0, 118]	93.0 [72.0, 114]
Missing	59 (2.1%)	438 (50.2%)	497 (13.3%)
HDL cholesterol, median [Q1, Q3], mg/dL^a^	47.0 [40.0, 56.0]	48.0 [39.0, 61.3]	47.0 [39.0, 57.0]
Missing	0 (0%)	420 (48.2%)	420 (11.3%)
Diabetes	582 (20.4%)	68 (7.8%)	650 (17.4%)
Hemoglobin A1c, median [Q1, Q3], %	5.40 [5.10, 5.70]	5.40 [5.10, 5.70]	5.40 [5.10, 5.70]
Missing	1 (0.0%)	383 (43.9%)	384 (10.3%)
Tobacco use			
Current	754 (26.4%)	296 (33.9%)	1050 (28.2%)
Former	963 (33.8%)	229 (26.3%)	1192 (32.0%)
Never	1136 (39.8%)	244 (28.0%)	1380 (37.0%)
Unknown	0 (0%)	103 (11.8%)	103 (2.8%)
Chronic kidney disease	549 (19.2%)	113 (13.0%)	662 (17.8%)
Estimated glomerular filtration rate, median [Q1, Q3], mL/min/1.73 m^2^	67.3 [55.4, 81.9]	68.9 [56.5, 84.7]	67.3 [55.4, 82.2]
Missing	0 (0%)	664 (76.1%)	664 (17.8%)
Obesity	585 (20.5%)	58 (6.7%)	643 (17.3%)
Body mass index, median [Q1, Q3], kg/m^2^	26.4 [23.3, 30.0]	24.7 [22.2, 28.1]	26.0 [23.1, 29.6]
Missing	34 (1.2%)	77 (8.8%)	111 (3.0%)
Prescribed medication classes			
Antihypertensive	816 (28.6%)	242 (27.8%)	1058 (28.4%)
Statin	1118 (39.2%)	128 (14.7%)	1246 (33.4%)
Aspirin	233 (8.2%)	51 (5.8%)	284 (7.6%)
Health system			
UCSF^b^	1034 (36.2%)	160 (18.3%)	1194 (32.1%)
SFDPH^c^	1819 (63.8%)	712 (81.7%)	2531 (67.9%)

^a^SI conversion factors: To convert cholesterol to mmol/L, multiply values by 0.0259

^b^*UCSF*, University of California, San Francisco

^c^*SFDPH*, San Francisco Department of Public Health

Among 2853 PWH with complete data, the median predicted 10-year absolute ASCVD risk using the PCE was 7.74% (IQR 3.74, 14.0) (Table 2) compared to 3.31% (IQR 1.88, 5.43) using PREVENT_full_. For comparison, the median risk was 4.26% (IQR 2.41, 7.26) using PREVENT_base_ and 5.62% (IQR 2.99, 10.5) using the revised PCE.

**Table 2:** Predicted 10-year Atherosclerotic Cardiovascular Disease Risk (*N*=2853)

	Pooled cohort equations	Revised pooled cohort equations	PREVENT_base_	PREVENT_full_
10-year ASCVD risk, median [Q1, Q3]	7.74 [3.74, 14.0]	5.62 [2.99, 10.5]	4.26 [2.41, 7.26]	3.31 [1.88, 5.43]

**Figure 1:**
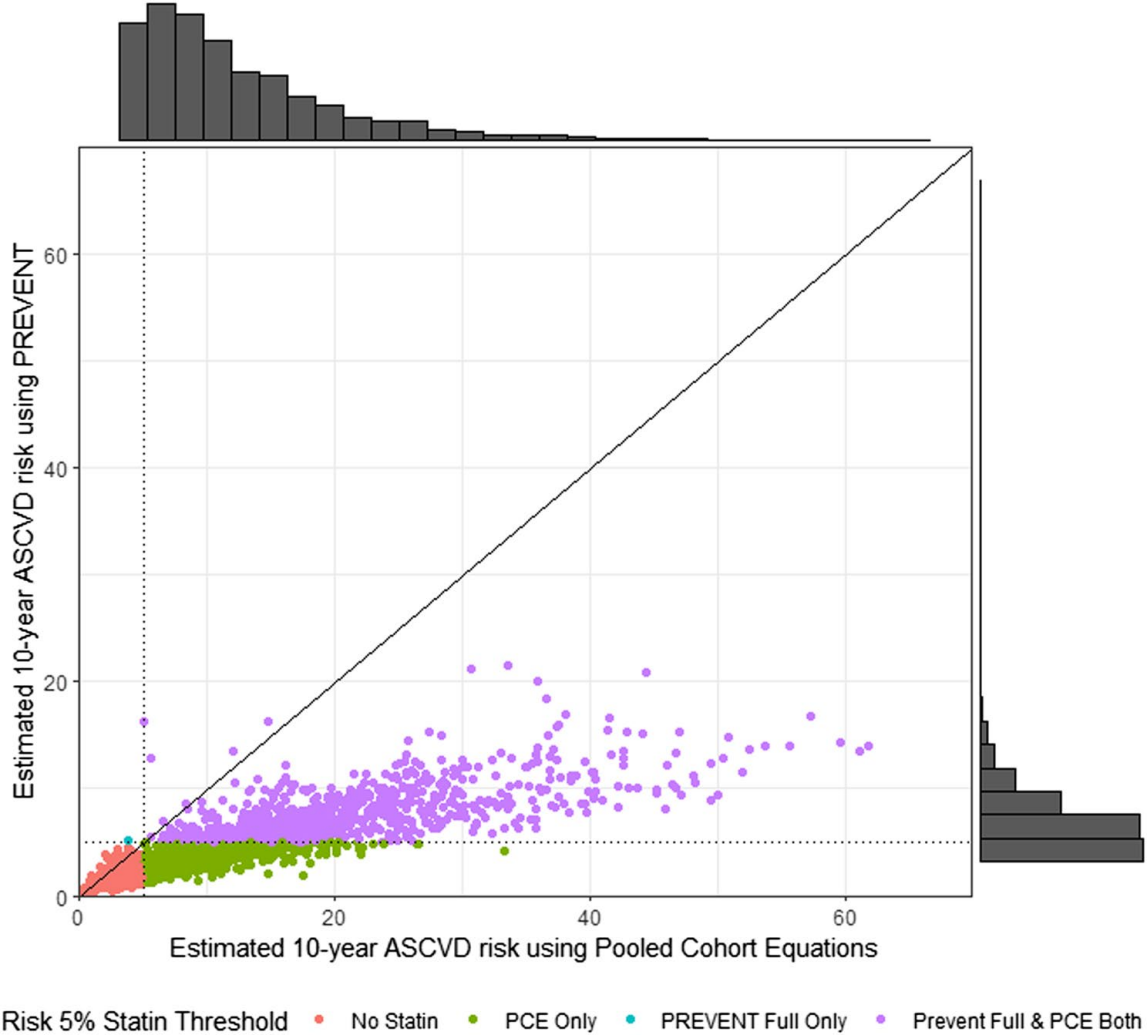
Comparison between predicted 10-year atherosclerotic cardiovascular disease risk using PCE and PREVENT. Each dot represents an individual’s predicted risk using the PCE on the x-axis and PREVENT_full_ on the y-axis, color coded to the impact on statin therapy; those in green would no longer be strongly recommended for statins with PREVENT but would have been with PCE. Histograms show the distribution of the two risk calculators; these also reveal the compression of predicted risk using PREVENT compared to PCE, which limits discrimination. Solid line (*y*=*x*) represents the expected average if both equations were calibrated the same. Dotted lines represent the 5% risk threshold for a strong statin recommendation.

Predicted risk is lower using PREVENT_full_ for 97% of the study population (Fig. 1). The mean difference in predicted risk (PCE-PREVENT_full_) is 6.35 percentage points (95% confidence interval 6.10, 6.59, *P*<0.0001). The mean difference between the revised PCE and PREVENT_full_ was less but still clinically relevant (3.94, 95% CI 3.76, 4.13, *P*<0.0001). There is also considerable compression of the range of predicted risk as shown by the histograms included in Fig. 1; the difference between the 1^st^ quartile and 3^rd^ quartile is 10.26 percentage points for the PCE compared to 3.55 percentage points for the PREVENT_full_. Results were similar when the complete population of 3725 individuals was included using multiple imputation for the missing data needed to calculate predicted risk (Supplemental Table 1).

Using the 10-year ASCVD risk of greater than or equal to 5% as the guideline-based threshold for a strong recommendation for statins, 58% fewer PWH would be strongly recommended for statins with PREVENT_full_ compared to the PCE (28.6% vs 67.3% of total population, Table 3, *P*<0.0001). In sensitivity analyses using the rPCE and PREVENT_base_, the proportions whose predicted risk was 5% or higher were 54.5% and 42.5%, respectively (Supplemental Tables 2a and 2b), with more agreement between the rPCE and PCE, as expected. The proportion of the primary prevention population specifically recommended for high-potency statins (predicted risk ≥20%) decreased from 13.0% with PCE to 0.1% with PREVENT_full_ (Table 4, *P*<0.0001).

**Table 3:** Statin Implications Using 5% Risk Threshold

PREVENT_full_				
		No statin	Statin	Total
Pooled cohort equations	No statin	933	1	934
	Statin	1104	815	1919 (67.3% recommended statin with PCE)
	Total	2037	816 (28.6% recommended statin with PREVENT_full_)	2853

**Table 4:** High-Potency Statin Implications Using 20% Risk Threshold

PREVENT_full_				
		No or Moderate Statin	High Intensity Statin	Total
Pooled Cohort Equations	No or Moderate Statin	2481	0	2481
	High Intensity Statin	368	4	372 (13.0% recommended high-potency statin with PCE)
	Total	2849	4 (0.1% recommended high-potency statin with PREVENT_full_)	2853

Currently, 1919 PWH meet the guideline recommendation for statins by PCE predicted risk, yet only 945 (49%) are prescribed statins. Among the 816 recommended for statins by their PREVENT_full_ risk, only 434 (53%) are prescribed statins.

The difference in predicted risk between the two equations varies by natal sex and race/ethnicity (Supplemental Figures 1 and 2). The median difference was greater for male than female individuals (− 4.84 vs − 1.36, *P*<0.0001, Supplemental Table 3). The median difference between the two predicted risks varied by race and ethnicity, with greater differences among Black individuals (P<0.0001, Supplemental Table 4).

To assess how these differences in predicted risk would impact health equity in recommendations for statins for primary prevention among PWH, we compared the proportion with a change in statin recommendation by sex and race/ethnicity. By sex, 130 (62.8%) of females recommended to be on a statin with PCE would no longer be using the PREVENT_full_ risk score, whereas 973 (56.9%) of males would no longer be recommended a statin (*P*=0.10). The proportion with a change in statin recommendation between the two equations was similar by race and ethnicity subgroups: 56.9% of white individuals, 56.8% of Black individuals, 58.8% of Hispanic individuals, and 58.8% of non-white, non-Black, and non-Hispanic individuals recommended a statin with PCE would no longer be recommended a statin with PREVENT_full_.

## DISCUSSION

In this study, we found that among PWH, the new PREVENT equations predict lower 10-year ASCVD risk compared to the PCE. The implication is that if the PREVENT equations are widely implemented in place of the PCE without recalibration or changes in current guidelines, many fewer PWH would be strongly recommended to be treated with statins for primary prevention of cardiovascular events. While we found differences in how the choice of calculator impacts the predicted risk by natal sex and race/ethnicity, the impact on statin recommendations varied less by these groups.

Our study demonstrates clinically meaningful differences in the predicted risk between the two equations but cannot assess which, if either, is calibrated correctly because we did not have longitudinal cardiovascular event outcomes available. Lower predicted risk is an intentional feature of the new equations by its developers given concerns that the PCE may overestimate risk in the contemporary general population.^17^ However, prior data suggest the PCE underestimates risk among PWH in multiple populations, especially among Black individuals and women in high-income countries such as the USA.^9–11,19^ Therefore, with lower predicted risk using the PREVENT equations, this underestimation is highly likely to be worse with PREVENT compared to the PCE. Future validation studies, ideally with adjudicated events, are urgently needed to assess the calibration of PREVENT (including comparison between base and full equations) among PWH overall and within specific subgroups prior to recommendation of their implementation specifically among PWH. With recalibration, these equations may be suitable for risk prediction for PWH.

Both calculators include versions of the same risk factors, such that they remain highly correlated with each other, but a few key differences are worth considering. Both include age, sex, current smoking, total cholesterol and HDL cholesterol, systolic blood pressure, diabetes, and hypertension treatment status. PREVENT_full_ swaps race for SDI and adds current statin use, estimated glomerular filtration rate, hemoglobin A1c, and urine protein creatinine ratio. One interesting implication is that if an individual moves to a different neighborhood, their predicted risk and therefore statin treatment recommendation may change. Whether excluding race from prediction models exacerbates disparities or promotes health equity remains controversial.

Our study sample with complete data had a high proportion with undetectable viral load (~85%) suggesting our results may be most applicable to those engaged in care with high levels of HIV control. However, only 65% with missing data were undetectable; our similar findings among those with imputed missing variables suggest that the difference in predicted risk between the two equations is probably independent of viral load or HIV control. Among those with ongoing HIV viremia, effective HIV treatment should be prioritized and can be done in parallel with initiation of statin therapy, if appropriate.

The major implication of our study is that implementation of the PREVENT equations would result in a dramatic reduction in the number of PWH strongly recommended for statins in the USA. We have illustrated the changes in predicted risk and statin therapy with a few hypothetical patient vignettes in Table 5. The clinical benefit of statins in preventing cardiovascular events in the general population has been well established in studies of more than 170,000 individuals.^20^ Given the known benefit of statin therapy now proven in PWH, reducing the number of PWH strongly recommended for statins appears to contradict the findings of benefit of pitavastatin found in REPRIEVE among individuals at low to intermediate predicted risk using the PCE.^3^ REPRIEVE did not find evidence of heterogeneity of treatment effect by predicted risk using the PCE, sex, race/ethnicity, or other risk factors. Nonetheless, the absolute benefit is greatest among those at highest risk, so the number needed to treat is lower among those with higher risk. The British HIV Society took a different approach from the US expert panel and recommended statins for all PWH over age 40 without the need for calculation of a risk score. In the setting of poor discrimination and calibration of risk calculators, this may be a reasonable approach.

**Table 5:** Change in Risk Prediction and Statin Recommendation for Sample Hypothetical Patients

	PCE		PREVENT_full_	
	Predicted 10-year ASCVD risk	Statin recommended	Predicted 10-year ASCVD risk	Statin recommended
55-year-old Black man with treated hypertension	12.4%	Yes	4.0%	No
65-year-old white woman with untreated hypertension (systolic blood pressure=145 mm Hg) who smokes	12.1%	Yes	4.8%	No
65-year-old Black woman with hyperlipidemia (total cholesterol=320 mg/dl)	11.1%	Yes	4.4%	No

### Limitations

Our study is a real-world electronic record-based cohort of individuals with at least one primary care or HIV clinic encounter and thus represents the population for whom a risk calculator may be used to target primary prevention strategies to prevent cardiovascular events, so it is not intended to be a population-based sample of all PWH. Misclassification of primary prevention status due to missing ICD-10 codes for prior ASCVD events or coding of “rule out diagnoses” is possible. To mimic clinical care, we used the most recent available lipid panels and creatinine, which were not necessarily on the same day as blood pressure measurement for all participants. Our dataset did not have urine protein creatinine ratios available, so we did not include this element in the PREVENT_full_ calculator although it should be incorporated if available. We did not compare the PREVENT equations with other risk calculators besides the PCE, such as Framingham, which has better calibration in some datasets,^6^ or the D:A:D calculator. As a San Francisco-based cohort, our study has limited representation of individuals who live in ZIP codes with low SDI, and we did not rescale the SDI as this is not specifically recommended by the PREVENT developers. Finally, we could not assess the calibration due to the cross-sectional nature of this dataset without longitudinal assessment of subsequent clinical ASCVD events.

## CONCLUSIONS

We found that the PREVENT equations predict much lower 10-year ASCVD risk compared to the PCE among PWH across two health systems in San Francisco. If implemented, PREVENT would result in many fewer PWH strongly recommended for statins. Given the known clinical benefits of statin use both in the general population and now PWH, the lower rates of statin use driven by the PREVENT equations could be detrimental in contributing to higher rates of cardiovascular events among PWH. The difference in predicted risk between the two equations varied by sex and race/ethnicity, which may have health equity implications. Given concerns that PREVENT may underestimate true ASCVD risk among PWH, further research to determine the calibration and discrimination of PREVENT among PWH should be done prior to implementation for PWH.

## Supplementary Information

Below is the link to the electronic supplementary material.

Supplementary file1 (DOCX 81 KB)

## Data Availability

Deidentified study data are available and stored at the Open Science Foundation at: https://osf.io/kq3az/?view_only=9482d60351124ef8b9a40ebc7a8aea49.
