# Supplementary material for: Prediction of Cardiovascular Risk Among People with HIV Using the PREVENT Equations Compared to the Pooled Cohort Equations
Source: J Gen Intern Med. 2025 Jun 6;41(1):64–72. doi: 10.1007/s11606-025-09642-z (PMC12855674; doi:10.1007/s11606-025-09642-z)
Supplement: Supplementary file 1 — Supplementary file1 (DOCX 81 KB) [file 11606_2025_9642_MOESM1_ESM.docx]

**Supplementary Information**

**Supplemental Table 1. Predicted Risk in Complete Case and Imputed Data**

|  | **Complete Case (N=2853)** | **All Data Including Imputed (N=3725)** |
| --- | --- | --- |
| **PCE** |  |  |
| Mean (SD) | 10.4 (9.16) | 10.13 (9.24) |
| Median [Q1, Q3] | 7.74 [3.74, 14.0] | 7.45 [3.49, 13.87] |
| **PREVENT_full_** |  |  |
| Mean (SD) | 4.03 (2.90) | 4.06 (2.97) |
| Median [Q1, Q3] | 3.31 [1.88, 5.43] | 3.33 [1.84, 5.46] |
| **Difference (PREVENT-PCE)** | | |
| Mean (SD) | -6.34 (6.74) | -6.85 (6.57) |
| Median [Q1, Q3] | -4.25 [-8.67, -1.73] | -4.84 [-9.30, -2.26] |

**Supplemental Table 2A. Differences in Statin Recommendations by revised PCE compared to PCE**

|  |  | **Revised PCE** | |  |
| --- | --- | --- | --- | --- |
|  |  | No Statin | Statin | Total |
| **Pooled Cohort Equations** | No Statin | 904 | 30 | 934 |
|  | Statin | 395 | 1524 | 1919  (67.3% recommended statin with PCE) |
|  | Total | 1299 | 1554  (55.5% recommended statin with RPCE) | 2853 |

**Supplemental Table 2b. Differences in Statin Recommendations by PREVENT_base_ compared to PCE**

|  |  | **PREVENT_base_** | |  |
| --- | --- | --- | --- | --- |
|  |  | No Statin | Statin | Total |
| **Pooled Cohort Equations** | No Statin | 925 | 9 | 934 |
|  | Statin | 715 | 1204 | 1919  (67.3% recommended statin with PCE) |
|  | Total | 1640 | 1213  (42.5% recommended statin with PREVENT_base_) | 2853 |

**Supplemental Table 3. Differences in Predicted Risk by Natal Sex**

|  | **Female (N=458)** | **Male (N=2395)** | **Overall (N=2853)** |
| --- | --- | --- | --- |
| **PCE** |  |  |  |
| Mean (SD) | 6.78 (7.68) | 11.1 (9.26) | 10.4 (9.16) |
| Median [Q1, Q3] | 4.17 [1.64, 8.85] | 8.41 [4.45, 14.8] | 7.74 [3.74, 14.0] |
| **PREVENT_full_** |  |  |  |
| Mean (SD) | 3.10 (2.61) | 4.21 (2.92) | 4.03 (2.90) |
| Median [Q1, Q3] | 2.50 [1.21, 4.19] | 3.50 [2.02, 5.64] | 3.31 [1.88, 5.43] |
| **Difference (PREVENT-PCE)** | | | |
| Mean (SD) | -3.68 (5.76) | -6.85 (6.79) | -6.34 (6.74) |
| Median [Q1, Q3] | -1.36 [-4.67, -0.296] | -4.84 [-9.30, -2.26] | -4.25 [-8.67, -1.72] |

**Supplemental Table 4. Differences in Predicted Risk by Race/Ethnicity**

|  | **White (N=1270)** | **Black (N=509)** | **Latino (N=689)** | **Other (N=385)** | **Overall (N=2853)** |
| --- | --- | --- | --- | --- | --- |
| **PCE** |  |  |  |  |  |
| Mean (SD) | 10.6 (8.93) | 13.5 (10.6) | 8.40 (8.12) | 9.13 (8.40) | 10.4 (9.16) |
| Median [Q1, Q3] | 8.16 [4.24, 14.1] | 10.6 [5.76, 18.0] | 5.80 [2.65, 11.6] | 6.57 [3.09, 12.6] | 7.74 [3.74, 14.0] |
| **PREVENT_full_** |  |  |  |  |  |
| Mean (SD) | 4.16 (2.83) | 4.58 (3.36) | 3.51 (2.69) | 3.77 (2.68) | 4.03 (2.90) |
| Median [Q1, Q3] | 3.48 [2.07, 5.57] | 3.88 [2.11, 6.23] | 2.66 [1.58, 4.70] | 3.17 [1.78, 4.98] | 3.31 [1.88, 5.43] |
| **Difference (PREVENT-PCE)** | | | | | |
| Mean (SD) | -6.42 (6.53) | -8.87 (7.98) | -4.89 (5.75) | -5.35 (6.27) | -6.34 (6.74) |
| Median [Q1, Q3] | -4.57 [-8.75, -1.96] | -6.46 [-12.4, -3.35] | -3.08 [-6.60, -0.961] | -3.47 [-7.29, -1.12] | -4.25 [-8.67, -1.72] |

**Supplemental Figure 1. Difference between PREVENT_full_ and PCE by Sex and Race/Ethnicity**


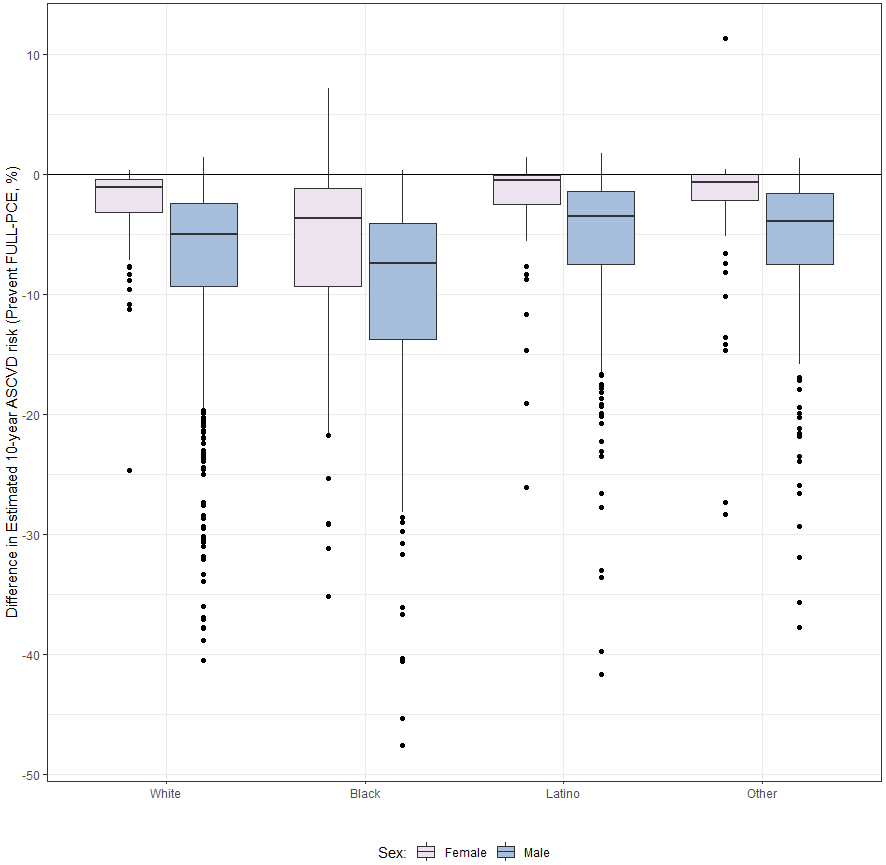


**Supplemental**

**Supplemental Figure 2. Graphical Comparison of Predicted Risk by PCE and PREVENT_full_ by Sex and Race/Ethnicity**


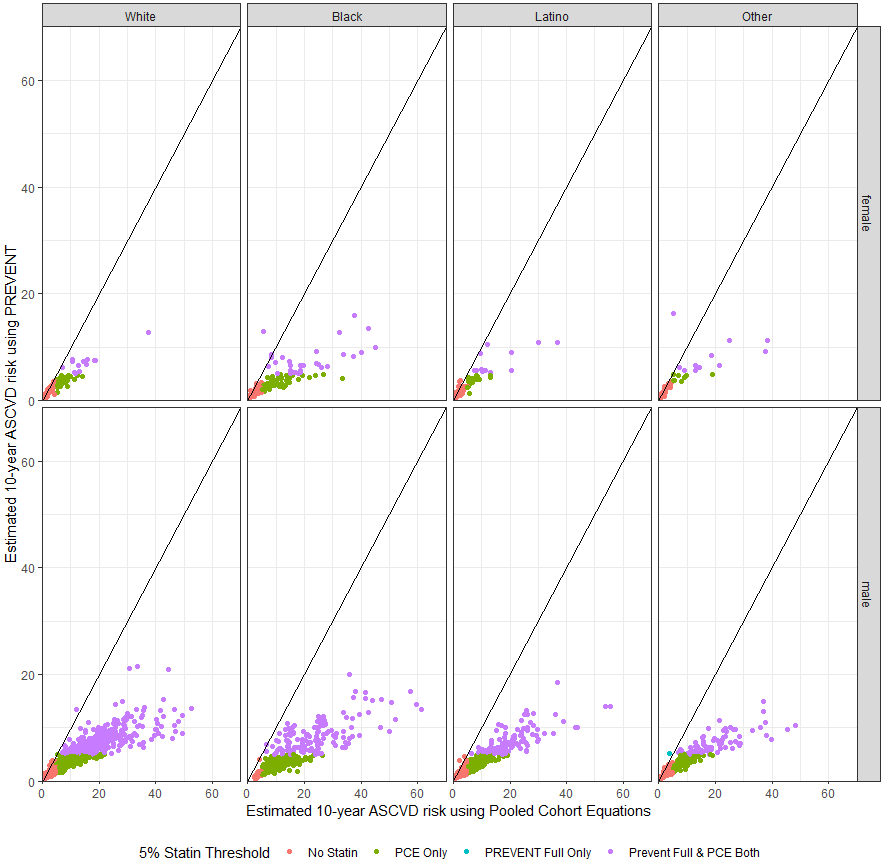


Supplemental Figure 2 Legend: While PREVENT_full_ predicts lower risk for all compared to the PCE, there is a greater difference between the two among males (bottom row) and among Black individuals (second column), which can be visualized as greater distance from the solid black lines and is most easily seen among the Black women subgroup where the slope is flatter. Solid diagonal lines represent equal predictions with the two equations.
